# Diverse Roles of JNK and MKK Pathways in the Brain

**DOI:** 10.1155/2012/459265

**Published:** 2012-02-08

**Authors:** Tokiwa Yamasaki, Hiroshi Kawasaki, Hiroshi Nishina

**Affiliations:** ^1^Department of Developmental and Regenerative Biology, Medical Research Institute, Tokyo Medical and Dental University, Tokyo 113-8510, Japan; ^2^Department of Molecular and Systems Neurobiology, Graduate School of Medicine, University of Tokyo, 7-3-1 Hongo, Bunkyo-ku, Tokyo 113-0033, Japan; ^3^Global COE Program “Comprehensive Center of Education and Research for Chemical Biology of the Diseases”, University of Tokyo, Tokyo 113-0033, Japan

## Abstract

The c-Jun NH_2_-terminal protein kinase (JNK) plays important roles in a broad range of physiological processes. JNK is controlled by two upstream regulators, mitogen-activated protein kinase kinase (MKK) 4 and MKK7, which are activated by various MAPKKKs. Studies employing knockout mice have demonstrated that the JNK signaling pathway is involved in diverse phenomena in the brain, regulating brain development and maintenance as well as animal metabolism and behavior. Furthermore, examination of single or combined knockout mice of *Jnk1, Jnk2,* and *Jnk3* has revealed both functional differences and redundancy among JNK1, JNK2, and JNK3. Phenotypic differences between knockouts of MKK4 and MKK7 have also been observed, suggesting that the JNK signaling pathway in the brain has a complex nature and is intricately regulated. This paper summarizes the functional properties of the major JNK signaling components in the developing and adult brain.

## 1. Introduction

The c-Jun NH_2_-terminal protein kinases (JNKs, also called stress-activated protein kinases (SAPKs)) are members of the evolutionarily conserved mitogen-activated protein kinase (MAPK) family [1, 2]. The JNK subfamily consists of three related genes: *Jnk1*, *Jnk2,* and *Jnk3*. In mammals, the JNK1 and JNK2 proteins are ubiquitously expressed, whereas JNK3 is found almost exclusively in the brain and testis. JNKs are activated by many types of external stress, including heat shock, UV irradiation, and inflammatory cytokines. JNKs phosphorylate numerous important substrates, including the transcription factors AP-1 and c-Jun, various apoptotic proteins, and microtubule-associated proteins (MAPs) [3–7]. Through phosphorylation of these substrates, JNKs regulate gene expression governing stress responses as well as the normal physiological processes of cell proliferation, apoptosis, differentiation, and cell migration.

 Activation of JNK is catalyzed by two kinases, mitogen-activated protein kinase kinase (MKK) 4 and MKK7 [8–10]. Although MKK4 and MKK7 are both dual-specificity Thr and Tyr kinases, previous studies of JNK activation have shown that MKK4 preferentially phosphorylates the Tyr residue of the TPY motif in the activation loop of JNKs, whereas MKK7 preferentially phosphorylates the Thr residue [11, 12]. The activation of MKK4 and MKK7 is mediated by various MAPKKKs, including mixed lineage protein kinases (MLKs), apoptosis signal-regulating kinases (ASKs) and dual leucine zipper kinase (DLK) [13]. In addition to regulation by these upstream kinases, the JNK signaling pathway is modulated by various scaffold proteins, including JNK-interacting protein (JIP) 1, JIP2, and JIP3 (also known as JNK/SAPK associated protein-1 (JSAP1)) [14–17]. These scaffold proteins assemble multienzyme complexes that involve a specific triad of a MAPKKK, a MAPKK, and a MAPK and provide an insulated physical conduit for signal transduction. The resulting linkage of these kinases forms a functional signaling module that performs transduction duties for a particular purpose.

**Figure 1:**
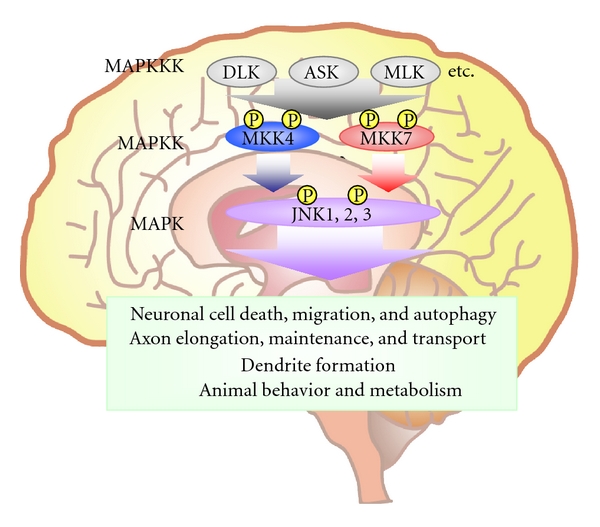
The JNK signaling pathway in the mammalian brain. A MAPKKK, such as DLK, ASK, or MLK, phosphorylates (P) and activates a MAPKK such as MKK4 or MKK7. An activated MAPKK in turn phosphorylates and activates a MAPK such as JNK1, JNK2, or JNK3. Activated JNKs then regulate various phenomena in the brain.

In most mammalian cell types, JNK activation is tightly controlled and employed at moderate levels in specific circumstances. In contrast, JNK signaling in the brain is highly and constitutively activated. Studies of gene knockout (KO) mice lacking JNKs or their upstream kinases have shown that this sustained activation is necessary to fulfil the diverse and essential roles that JNK signaling plays in the brain (Figure 1). This paper summarizes the phenotypes of these mutant animals and discusses what they reveal about the regulation and various functions of JNK and MKK signaling in the brain.

## 2. Brain Phenotypes of JNK Knockout Mice

### 2.1. *Jnk1/2* DKO Mice: JNK1/2 Induces Programmed Cell Death during Early Brain Development

Knockout mice lacking one, two, or all JNK isoforms have been generated over the past decade, and their diverse phenotypes have been reported (Table 1). Importantly, embryonic lethality is not caused by the single KO of any *Jnk* gene. Two strains of double KO mice, *Jnk1^−/−^Jnk3^−/−^*(*Jnk1/3 DKO*) and *Jnk2^−/−^Jnk3^−/−^*(*Jnk2/3 DKO*), are viable, but *Jnk1* ^−/−^ *Jnk2^−/−^* (*Jnk1/2 DKO*) animals exhibit early embryonic lethality.

During early brain development in mice, the neural folds emerge at embryonic day (E) 7.5 in the cephalic region, close over at E8.5–E9 (neural tube closure), and form the neural tube. The *Jnk1/2* DKO mouse is lethal at E11.5 due to dysregulation of apoptosis in the neural tube [18, 19]. Specifically, there is a marked reduction in cell death in the lateral edges of the hindbrain prior to neural tube closure. In contrast, increased apoptosis and caspase activation are found in the mutant forebrain, leading to precocious degeneration. Interestingly, about 25% of *Jnk1^−/−^Jnk2^+/−^* fetuses display excencephaly that is likely the result of failed neural tube closure, whereas* Jnk1^+/−^Jnk2^−/−^* mice are normal [19]. These results suggest that JNK1 and JNK2 are redundant regulators of programmed cell death during early brain development and that JNK1 is dominant in this particular function of the JNK signaling pathway.

### 2.2. *Jnk1^−/−^* Mice: JNK1 Is Required for Neuronal Migration, Dendrite Formation, and Axon Maintenance during Later Brain Development

After neural tube formation at E8.5–E9, three of its vesicles give rise to various specialized regions of the brain. Neuronal progenitors proliferate and generate immature neurons. These cells subsequently migrate from the proliferative zones to their final positions, where they extend neurites. For example, in the developing cortex, neural progenitors proliferate from E11 to E17 then generate immature neurons that migrate and form the cortical layers, the cortical plate, subplate, intermediate zone, and ventricular zone. The extension and maturation of neurites by these cells then continues during the remaining embryonic and postnatal stages. *Jnk1^−/−^* embryos display thicker cortical plates and thinner ventricular zones than wild-type animals [20]. *Jnk1^−/−^* embryos also show abnormally accelerated migration of cortical neurons (radial migration), a process whose precise regulation is required for the correct formation of cortical layers. These findings indicate that JNK1 regulates the rate of neuronal migration during cortical development.

Other studies have shown that JNK1 also regulates neurite formation and maintenance. Neurons extend two types of neurites: dendrites and axons. Compared to wild-type mice, dendrites in the cortex and cerebellum of *Jnk1^−/−^* mice are shorter and have more processes [21], indicating that JNK1 plays an important role in defining dendritic architecture during brain development. In contrast, axon formation is not affected by JNK1 disruption. Axon tracts such as the corpus callosum and anterior commissure appear normal in *Jnk1^−/−^* mice until postnatal day (P) 6. From P6-P12, however, the anterior commissure degenerates, demonstrating that JNK1 is required for axon maintenance [22].

One of the molecular mechanisms mediating JNK pathway functions during brain development and maintenance is the phosphorylation of MAPs such as MAP1B, MAP2, and superior cervical ganglion 10 (SCG10) [20–22]. MAPs bind to microtubules (MTs) and modulate their stability and structure. Importantly, the phosphorylation of MAPs by one of several protein kinases (including JNK) regulates the binding of these enzymes to MTs and thus the regulation of MT modification. In *Jnk1^−/−^* brain, the phosphorylation of MAP1B, MAP2, and SCG10 is reduced. Moreover, the introduction of a mutated SCG10 protein that mimics the JNK1-phosphorylated form restores normal neuronal migration in the brains of *Jnk1^−/−^* embryos [20]. These results demonstrate that, in addition to regulating radial migration, dendrite formation, and axon maintenance in the developing brain, JNK1 controls MT structure through MAP phosphorylation.

### 2.3. *Jnk2^−/−^* and *Jnk3^−/−^* Mice: JNK2 and/or JNK3 Are Required for Neuronal Cell Death Induced by Neuronal Stresses in Adult Brain

The redundancy of many JNK functions is highlighted by the fact that *Jnk2^−/−^* and *Jnk3^−/−^* single KO mice show none of the defects observed in *Jnk1^−/−^* mice. However, *Jnk2^−/−^* and *Jnk3^−/−^* mice do show alterations in neuronal stress-induced neuronal cell death that are rarely observed in *Jnk1^−/−^* mice, implying that each JNK enzyme has a unique function. For example, 1-methyl-4-phenyl-1,2,3,6-tetrahydropyridine (MPTP) is a neurotoxin whose administration induces dopaminergic cell demise and so results in symptoms that replicate most of the neuropathological hallmarks of Parkinson's disease [23]. *Jnk2^−/−^* or *Jnk3^−/−^* mice (but not *Jnk1^−/−^* mice) display resistance to MPTP-induced neuronal cell death *in vivo* [24]. Moreover, this resistance to MPTP is enhanced by double mutation of *Jnk2* and *Jnk3*, indicating that JNK2 and JNK3 play partially overlapping roles in this process. *Jnk3^−/−^* mice also display resistance to kainic acid-induced cell death (excitotoxicity-induced apoptosis) and ischemia-induced cell death *in vivo* [25–27], as well as reduced susceptibility to apoptosis induced by the Alzheimer's disease-related protein beta-amyloid *in vitro* [28]. Finally, *Jnk3^−/−^* mice intrastriatally injected with 6-hydroxydopamine, which provokes the death of dopaminergic neurons, show a transient prolongation of dopaminergic neuron survival in the substantia nigra compacta compared to injected control mice [29]. These results suggest that the physiological function of both JNK2 and JNK3 in the brain is to induce apoptosis in response to neuronal stress.

### 2.4. Conditional *Jnk1^−/−^* Mice: JNK1 Activity in Adult Brain Regulates Animal Metabolism

Intact JNK signaling in the adult brain is required for normal animal metabolism. A high-fat diet induces JNK activation in the hypothalamus and pituitary, which regulate body weight control, glucose homeostasis, and secretion of hormones. Conditional KO (cKO) mice have been generated in which *Jnk1* expression is controlled by Nestin-Cre, which induces Cre recombinase expression in neural stem cells [30]. Upon high-fat feeding, *Jnk1^flox/flox^ Nestin-Cre* mice exhibit increased insulin sensitivity (compared to wild-type controls) both in the CNS and in peripheral tissues, improved glucose metabolism and protection from hepatic steatosis and adipose tissue dysfunction. *Jnk1^flox/flox^ Nestin-Cre* mice also display reduced somatic growth and altered secretion of growth hormone (GH), insulin-like growth factor (IGF), and thyroid hormones. JNK activity in the brain is thus required for normal metabolism.

JNK signaling in the brain is also involved in the control of feeding. The hypothalamus governs food intake in response to nutrient status, cytokines, and hormones. *Jnk1^−/−^* mice exhibit enhanced food intake and weight gain upon hypothalamic administration of glucocorticoid [31]. Moreover, JNK1 disruption increases mouse sensitivity to the anorexigenic effects of hypothalamic insulin administration. These data show that JNK signaling in the brain regulates not only hormone secretion and peripheral metabolism but also feeding and thus contributes to the maintenance of energy homeostasis. It remains unclear whether JNK2 and/or JNK3 are also involved in these functions, but improved insulin resistance has been reported for both *Jnk1^+/−^Jnk2^−/−^* mice and *Jnk1^−/−^* mice fed a high-fat diet [32, 33]. These results suggest that JNK2 may be involved in the central and/or peripheral regulation of glucose homeostasis. Analyses of cKO mice lacking *Jnk1* and/or *Jnk2* will resolve this issue.

## 3. Brain Phenotypes of MKK Knockout Mice

Because JNK isoforms are required for brain development, and MKK4 and MKK7 activate all JNK isoforms, it is not surprising that disruption of *Mkk4 *and/or *Mkk7* results in severe defects in mouse development. *Mkk4^−/−^Mkk7^−/−^* (*Mkk4/7 DKO*) mice die at E8.5 before neural tube formation, and both *Mkk4^−/−^* and *Mkk7^−/−^* single KO mice are embryonic lethal at around E11.5 due to impaired liver formation [9, 34–36]. To circumvent this limitation, the functions of MKK4 and MKK7 during brain development have been investigated using Nestin-Cre cKO mice lacking MKK4 and/or MKK7. Analyses of these mutants have proven very helpful in overcoming the conundrum posed by the redundancy of JNK isoforms.

### 3.1. Mkk4^*flox/flox*^ Nestin-Cre Mice: MKK4 Is Required for Neuronal Migration and Axon Maintenance in the Developing Brain

In *Mkk4^flox/flox^ Nestin-Cre* mice, total JNK activation in the brain is reduced to 20% of normal but these animals are not embryonic lethal and dysregulated apoptosis is not observed [37]. At birth, *Mkk4^flox/flox^ Nestin-Cre* mice are indistinguishable from their control littermates, but the mutants stop growing a few days later and die at around 3 weeks of age. The brains of *Mkk4^flox/flox^ Nestin-Cre* mice display misaligned Purkinje cells in the cerebellum and delayed radial migration in the cerebral cortex. In their commissural axon tracts, axonal degeneration is observed not only in the anterior commissure, the site of a similar defect in *Jnk1^−/−^* brain [22], but also in the corpus callosum. At the molecular level, hypophosphorylation of MAP1B is observed in *Mkk4^flox/flox^ Nestin-Cre* mice, suggesting that dysregulation of MT dynamics is involved in these phenotypes. The altered neuronal cell migration and axon maintenance observed in *Mkk4^flox/flox^ Nestin-Cre* mice are also seen in *Jnk1^−/−^* mice, but these phenotypes are less severe in the latter. Thus, MKK4 is a regulator of radial migration and axon maintenance in the brain, and MKK4's effects are mediated not only by JNK1 but also by JNK2 and/or JNK3.

### 3.2. Mkk7^*flox/flox*^ Nestin-Cre Mice: MKK7 Is Required for Neuronal Migration and Axon Elongation in Developing Brain

Our group has generated *Mkk7^flox/flox^ Nestin-Cre* mice [38]. Unlike *Mkk4^flox/flox^ Nestin-Cre* mice, which survive until age 3 weeks, *Mkk7^flox/flox^ Nestin-Cre* mice die at birth without breathing. Like *Mkk4^flox/flox^ Nestin-Cre* mice, JNK activation is reduced to 20% of normal in the developing brain of *Mkk7^flox/flox^ Nestin-Cre* mutants, and a delay in neuronal migration in the cerebrum is observed. However, other phenotypes do not overlap between *Mkk7^flox/flox^ Nestin-Cre* and *Mkk4^flox/flox^ Nestin-Cre* mice (Table 2). At E18.5, *Mkk7^flox/flox^ Nestin-Cre* mice display enlarged brain ventricles, diminished striatum, decreased forebrain axon tracts, and reduced corticofugal axons; none of these defects has been found in *Mkk4^flox/flox^ Nestin-Cre* mice. In addition, ultrastructural alterations such as abnormal accumulations of filamentous structures and autophagic vacuoles are observed in *Mkk7^flox/flox^ Nestin-Cre* brain but not in *Mkk4^flox/flox^ Nestin-Cre* brain. Thus, *Mkk7* has unique functions in the developing brain that differ from those of MKK4.

**Figure 2:**
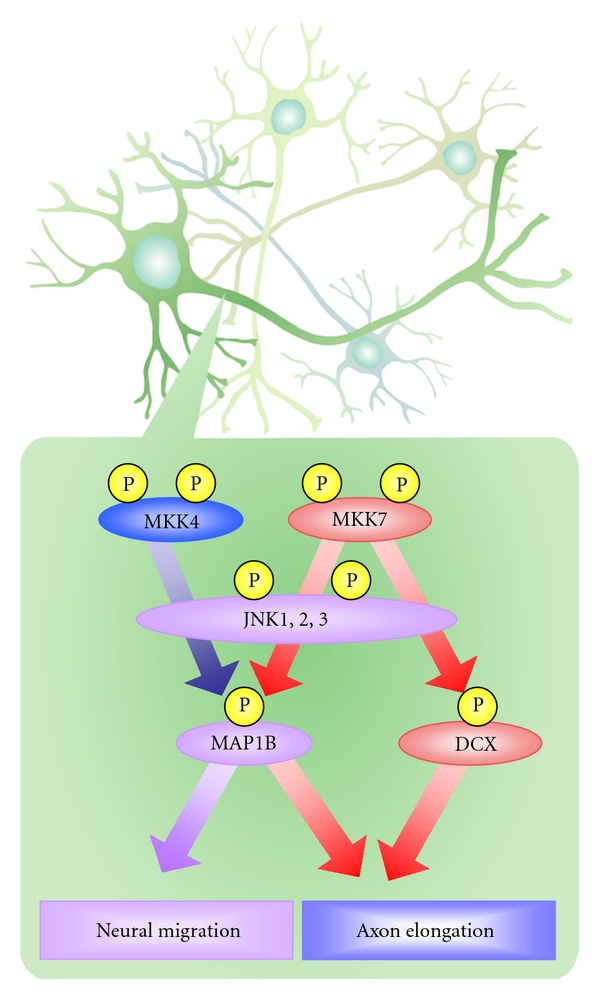
MKK4 and MKK7 have different functions in the developing brain. MKK4 and MKK7 both activate JNKs, which phosphorylate MT-associated proteins such as MAP1B and DCX. Activated MAP1B and DCX regulate neuronal migration and axon elongation in the developing brain. However, JNK activated by MKK4 regulates radial migration but not axon elongation, whereas JNK activated by MKK7 controls both radial migration and axon elongation. These differences between MKK7 and MKK4 functions also appear at the molecular level. The phosphorylation of MAP1B requires that JNK be activated by both MKK4 and MKK7. However, the phosphorylation of DCX requires JNK activation only by MKK7 and not by MKK4.

Differences between MKK7 and MKK4 functions also appear at the molecular level. In *Mkk4^flox/flox^ Nestin-Cre* brain, phosphorylation levels of MAP1B are reduced but DCX phosphorylation is not altered. In contrast, phosphorylation levels of both MAP1B and DCX are decreased in *Mkk7^flox/flox^ Nestin-Cre* brain, suggesting that the MKK7-JNK and MKK4-JNK signaling modules in this organ are not identical. In line with this hypothesis, the scaffold protein JIP1 binds to JNK, MKK7, and DCX but not to MKK4. We therefore propose that differences in scaffold proteins and/or substrates involved in the MKK7-JNK versus MKK4-JNK pathways could cause the phenotypic divergence observed between *Mkk7^flox/flox^ Nestin-Cre *and *MKK4^flox/flox^ Nestin-Cre* mice (Figure 2).

## 4. Brain Phenotypes of MAPKKK Knockout Mice

Compared with MAPKKs, MAPKKKs comprise a much larger family of related proteins. Indeed, at least 12 MAPKKKs have been identified as regulating various steps of the JNK signaling pathway [13]. This multiplicity of related functions suggests that each MAPKKK has a specific spatiotemporal role in controlling JNK signaling. However, the precise mechanisms by which most MAPKKKs regulate JNKs and their biological roles in the brain have yet to be fully elucidated. We summarize below evidence supporting the importance of two MAPKKKs, DLK, and ASK1, in the JNK signaling pathway in the brain.

### 4.1. *Dlk^−/−^* Mice: DLK Is Required for Axon Elongation and Neuronal Migration in Developing Brain

DLK is known to be critical in the developing mammalian brain.* Dlk^−/−^* mice die perinatally, with no homozygous mutant surviving until weaning. *Dlk^−/−^* mice display retarded radial migration and impaired fiber tract development by neocortical pyramidal neurons in the cerebrum [40]. Direct and quantitative analysis of pyramidal neuron radial migration using slice culture and a time-lapse imaging system has revealed that *Dlk* disruption affects acceleration around the cortical subplate. Furthermore, *in vitro* culture of *Dlk^−/−^* neurons has shown that DLK is involved in the establishment of neuronal polarity and regulates the MT dynamics driving the transition of stage 1 (nonpolar) neurons to stage 2 (multipolar) neurons and then the transition of stage 2 to stage 3 (axon-forming) neurons [41]. These results demonstrate that DLK regulates radial migration and axon formation during brain development. However, a reduction of only 30% in total phosphorylated JNK is observed in *Dlk^−/−^* brain, indicating that other MAPKKKs must be involved in JNK activation in this organ. The identities of these enzymes are under active investigation.

### 4.2. *Ask1^−/−^* Mice: ASK1 Drives Neuronal Cell Death in Adult Brain Following Ischemia or Neurodegeneration and Regulates Animal Behavior

ASK1 is well known as a proapoptotic MAPKKK that is involved in responses to diverse stresses and activates both the JNK and p38 signaling pathways [42]. *Ask1^−/−^* mice are born at the expected Mendelian frequency and show no developmental abnormalities as determined by histological analysis [43]. Retinal ganglion cells of *Ask1^−/−^* mice do not readily undergo ischemia-induced apoptosis *in vivo* [44], and *Ask1^−/−^* primary neurons display resistance to neuronal cell death triggered by polyglutamine *in vitro* [45]. In mice transgenic for a mutation of Cu/Zn-superoxide dismutase, which serve as a model of human amyotrophic lateral sclerosis, deletion of ASK1 mitigates motor neuron loss and extends mouse lifespan [46]. These data indicate that ASK1's proapoptotic functions extend to neurons *in vivo*. ASK1 also has nonapoptotic functions in the brain, since *Ask1^−/−^* mice exhibit temporary hyperactivity in an open-field test [47]. Interestingly, this hyperactivity is specific to the novel environment, with *Ask1^−/−^* mice displaying normal activities in the familiar field. *Ask1^−/−^* mice also show impaired novelty preference at 24 hours after training but superior performance on the rotarod test. These results demonstrate that ASK1 is involved in locomotor activity, novelty preference, and motor coordination requiring dopaminergic transmission.

## 5. Other Evidence Supporting Roles for JNK Signaling in the Nervous System

### 5.1. Regulation of Axonal Transport

In *Drosophila*, an absence of the function of either Bsk (*Drosophila* JNK) or Hep (*Drosophila* MKK7) causes a failure of lateral epithelial cells to stretch such that the embryo develops a hole in the dorsal cuticle [48–50]. These data indicate that the MKK7-JNK signaling pathway mediates cell migration in *Drosophila* and regulates dorsal closure during early morphogenesis.

**Figure 3:**
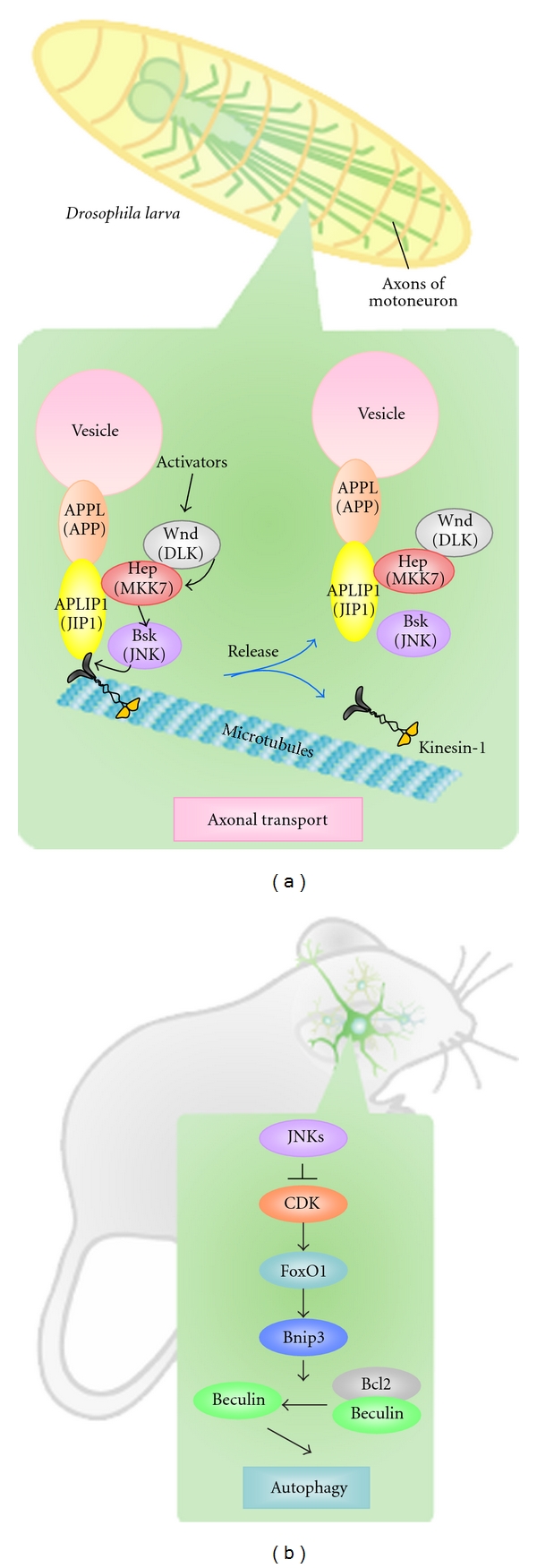
The JNK signaling pathway regulates axonal transport and autophagy. (a) Model of how the JNK signaling pathway controls axonal transport in *Drosophila*. Axonal transport is driven along MT by kinesin-1, which binds to the vesicles that make up its cargo through APLIP1 (*Drosophila* JIP1) and APPL (APP). This process is driven by Wnd (DLK), which is activated by unknown upstream signals, and phosphorylates Hep (MKK7). Activated Hep then phosphorylates and activates Bsk (JNK), which then directly or indirectly modifies the linkage complex and causes APLIP1 and the cargo to dissociate from kinesin-1. (This figure is excerpted from [39] with some modifications.) (b) Model of the regulation of neuronal autophagy in mice. In normal neurons, constitutively activated JNKs suppress CDK-induced FoxO1 activation, preventing autophagy. When all three of JNK1, JNK2, and JNK3 are disrupted, CDK-mediated FoxO1 activation increases Bnip3 expression. High levels of Bnip inhibit the binding of Beclin1 to Bcl2, and this freshly released Beclin1 induces autophagy.

Axonal transport in *Drosophila* is driven along MT by the kinesin motor system, and APLIP (*Drosophila* JIP1) is known as a “cargo linker” that joins kinesin-1 to various vesicle proteins such as the *Drosophila* equivalent of the Alzheimer's APP protein [51]. *Drosophila* axonal transport is believed to be regulated by the JNK signaling pathway because mutation of Wnd (*Drosophila* DLK), Hep, or Bsk results in the abnormal accumulation of synaptic vesicles in the nerves of third instar larvae [39]. Mutation of Wnd or Hep also disrupts the binding of kinesin-1 to APLIP1. Thus, the JNK signaling pathway regulates the attachment of APLIP1 to kinesin-1 in *Drosophila*, ensuring that the dissociation of kinesin-1 from its vesicle protein cargo occurs at the appropriate position (Figure 3(a)).

The involvement of the JNK signaling pathway in axonal transport has also been reported in mammals. JNK can be activated by the pathogenic polyglutamine-containing Huntingtin protein associated with human Huntington's disease. Fast axonal transport is then inhibited because JNK3 directly phosphorylates the kinesin-1 motor domain [52]. It remains to be determined how extensively this molecular mechanism is conserved between *Drosophila* and mammals.

### 5.2. Regulation of Neuronal Autophagy

Conditional *Jnk1, Jnk2, Jnk3* triple knockout mice (*Jnk1/2/3 cTKO* mice) have been generated to eliminate the problem of functional redundancy among JNK1, 2, and 3 [53]. These animals were created by crossing *Jnk1 flox* mice with *Jnk2^−/−^*, *Jnk3^−/−^*, and Cre-expressing strains. *Jnk1/2/3 cTKO Nestin-Cre* mice are embryonic lethal at an early stage, but *Jnk1/2/3 cTKO Pcp2-Cre* mice, which express Cre in Purkinje cells, are viable. While numerous abnormal phenotypes have been reported for *Jnk1/2/3 cTKO Pcp2-Cre* mice, including axon hypertrophy, abnormal mitochondrial transport, and prolonged cellular lifespan in culture, the most striking anomaly observed has been increased neuronal autophagy [53]. Results to date indicate that, in neurons, the JNK signaling pathway suppresses autophagy, whereas, in nonneuronal cells, JNK signaling either induces autophagy or serves as an effector of autophagy-associated cell death [54, 55]. Although the JNK substrate leading to autophagy has yet to be identified, it has been shown that increased autophagy in* Jnk1/2/3 cTKO* neurons is mediated by FoxO1 and not by an mTORC1-dependent mechanism [53]. The combined disruption of JNK1, JNK2, and JNK3 increases Bnip3 expression through FoxO1 activation that is mediated by CDK (Figure 3(b)). Bnip3 inhibits Beclin1-Bcl2 binding and induces Beclin1 release, which in turn triggers autophagy.

## 6. Conclusion and Future Perspectives

Studies of KO mice lacking JNKs, MAPKKs or MAPKKKs, have revealed that the JNK signaling pathway is involved in diverse roles in the brain, including induction of neuronal cell death, radial migration, neurite formation, metabolism regulation, and behavioral control. Twelve MAPKKKs, two MAPKs, and three JNKs can be combined in triads with specific scaffold proteins to form a large array of JNK signaling modules. These various modules are believed to facilitate JNK participation in specific biological functions. Indeed, the difference in phenotypes displayed by *Mkk4^flox/flox^ Nestin-Cre *and *Mkk7^flox/flox^ Nestin-Cre* mice reinforces the notion that there are distinct JNK signaling modules that may be MKK4 dependent or MKK7 dependent. Thus, to truly elucidate the JNK signaling network, it will be necessary to investigate JNK signaling at every step of its hierarchy: MAPKs, MAPKKs, and MAPKKKs.

The brain contains many different cell types, including various classes of neurons, astrocytes, and oligodendrocytes. To date, cell type-specific analyses of JNK signaling have not been performed. Future reviews will no doubt focus on the use of cell type-specific cKO mice to further dissect the striking reach of JNK signaling in normal and abnormal physiology.

## Figures and Tables

**Table 1:** Phenotypes of JNK knockout mice.

Mouse model	Phenotypes	References
*Jnk1^−/−^*	Accelerated radial migration Shorter dendrites with increased branching Degeneration of anterior commissure Enhanced glucocorticoid- or insulin-induced food intake	[20–22, 31]

*Jnk2^−/−^*	Resistance to MPTP-induced neuronal cell death	[24]

*Jnk3^−/−^*	Resistance to MPTP-induced neuronal cell death Resistance to kainic acid-induced neuronal cell death Resistance to ischemia-induced neuronal apoptosis Resistance to 6-hydrozydopamine-induced neuronal apoptosis	[24–27, 29]

*Jnk1^−/−^ Jnk2^−/−^*	Embryonic lethal at E11.5 Defective neural tube closure Dysregulated apoptosis in brain (increased in forebrain, reduced in hindbrain)	[18, 19]

*Jnk2^−/−^ Jnk3^−/−^*	Resistance to MPTP-induced neuronal cell death	[24]

*Jnk1^flox/flox^* *Nestin-Cre*	Animal protected from diet-induced glucose intolerance and insulin resistance Reduced serum IGF-1 and GH Increased serum thyroid hormones	[30]

*Jnk1^flox/flox^Jnk2^−/−^* *Jnk3^−/−^* *Nestin-Cre*	Early embryonic lethal	[53]

*Jnk1^flox/flox^Jnk2^−/−^* *Jnk3^−/−^* *Pcp2-Cre*	Loss of dendritic arborization Axon hypertrophy Increased autophagic vacuoles	[53]

**Table 2:** Comparison of phenotypes of *Mkk4* ^*flox/flox*^ *Nestin-Cre* and *Mkk7* ^*flox/flox*^ *Nestin-Cre mice*.

Phenotype	*Mkk4^flox/flox^ Nestin-Cre* [37]	*Mkk7^flox/flox^ Nestin-Cre* [38]
Neuronal JNK activity	Suppressed	Suppressed
Age of lethality	Around 3 weeks old	At birth
Brain ventricle size at E18.5	Not reported	Enlarged
Striatum	Not reported	Reduced
Axon tracts (E18.5)	Unaffected	Greatly reduced
Axon tracts (postnatal)	Less fasciculated	Not determined
Intermediate filaments	Not reported	Accumulate in axons
Autophagic vacuoles	Not reported	Accumulate
JNK phosphorylation in axons	Not reported	Suppressed
L1-positive axons (E18.5)	Unaffected	Reduced
TAG-1-positive axons (E18.5)	Not reported	Reduced
Apoptosis	Unaffected	Unaffected
Cortical layer markers	Not reported	Expressed
Radial migration	Delayed	Delayed
Phosphorylation of c-Jun	Suppressed	Suppressed
Phosphorylation of NF-H	Suppressed	Suppressed
Phosphorylation of MAP1B	Suppressed	Suppressed
Phosphorylation of DCX	Unaffected	Suppressed
